# Correlation between Motorcycle Riding and Erectile Dysfunction on Online Motorcycle Taxi Drivers

**DOI:** 10.5152/tud.2023.22174

**Published:** 2023-03-01

**Authors:** Ricky Adriansjah, Ahlan Syahreza, Bambang Sasongko Noegroho, Tjahjodjati Tjahjodjati, Kuncoro Adi, Jupiter Sibarani, Zola Wijayanti, Teguh Marfen Djajakusumah

**Affiliations:** 1 Department of Urology, Padjadjaran University, Faculty of Medicine, Bandung, Indonesia; 2 Department of Vascular Surgery, Padjadjaran University, Bandung, Indonesia

**Keywords:** Motorcycle, online motorcycle driver, erectile dysfunction, IIEF-5

## Abstract

**Objective:**

Erectile dysfunction is a condition in which the patient is unable to achieve or maintain a sufficient erection for sexual intercourse. Transportation usage was believed to have a higher risk of erectile dysfunction. This study aimed to assess the correlation between activity and severity of erectile dysfunction among online motorcycle taxi drivers who use motorbikes.

**Materials and methods:**

This research uses an observational analytic method with a cross-sectional approach with primary data collection from the respondents who work as online motorcycle drivers from January 2021 to March 2021. Data analyses were conducted using Mann–Whitney and Spearman statistical test.

**Results:**

A total of 149 respondents met the inclusion and exclusion criteria contained in this study. It was found that the prevalence of erectile dysfunction in this study was 57.7%. There was a significant difference in the distance covered (km) by online motorcycle taxi drivers who experienced erectile dysfunction compared to those who did not have erectile dysfunction (*P* = .050). In addition, there was a significant difference in length of work (year) among online motorcycle taxi drivers who experienced erectile dysfunction (*P* = .045).

**Conclusion:**

There was a significant difference in erectile dysfunction incidence based on the distance covered per day and length of work in online motorcycle taxi driver. No significant difference was found in the incidence of erectile dysfunction based on the length of motorbike drive per day. The more distance covered (km) and length of work (year) and the more severe the erectile dysfunction are based on international index of erectile function-5 score.

**Main Points**

- Two-wheeled vehicles such as bicycles or motorbikes can be associated with the risk of developing erectile dysfunction (ED).
- This study shows the prevalence of 57.7% of ED in online motorcycle taxi drivers.
- Distance traveled per day using motorcycle and the duration of work correlate to the severity of ED.

## Introduction

Erectile dysfunction (ED) is a condition in which the patient is unable to achieve or maintain an erection sufficient for sexual intercourse. The incidence of ED is currently estimated at around 25.9 cases per 1000 men per year based on a study conducted in Massachusetts.^1^ The degree of ED can be measured by the international index of erectile function (IIEF-5). The degree of ED is divided into mild, mild–moderate, moderate, or severe. The causes of ED may vary, ranging from vascular, endocrine, and neurological to psychological causes.^2^

The use of transportation, especially 2-wheeled vehicles such as bicycles or motorbikes, can be associated with the risk of developing ED in men. Individuals who cycle have an approximately 1.75-fold greater risk of developing ED compared to the general population. In cyclists who cover a distance of 540 km, about 22% had penile numbness and 13% had ED.^2^ The possible mechanism involved in the occurrence of ED in men who ride 2-wheeled vehicles is due to compression of the perineum leading to occlusion of the pudendal artery and pudendal nerve. Nervous disorders can also be found in people who have a history of riding a bicycle more than 3 hours per week.^3,4^

Motorcycles are one of the short-distance transportation in Indonesia. Motorcycles have a fairly rapid growth development. Data from the Central Statistics Agency in 2018 show that the number of motorcycles in Indonesia reached 106 million.^5^ To date, there has been no research examining the incidence of ED and its relation to motorcycle use in Indonesia. Considering that motorbikes are one of the main means of transportation in Indonesia and online motorbike taxis use motorbikes in their daily lives, this study aims to assess the correlation between activity and severity of ED in online motorbike taxi drivers who use motorbikes.

## Materials and Methods

This research is an observational analytic study with a cross-sectional approach. This study aims to determine the correlation between online motorcycle taxi drivers who ride motorcycles and the degree of ED. Data will be taken from online motorcycle taxi drivers who meet the criteria. Subjects who meet the criteria will be measured for various variables to be studied and the data will be analyzed to find a correlation between online motorcycle taxi drivers who ride motorbikes and the severity of ED. Ethics for this research was approved on March 30. 2021, with ethical approval number: 230/UN6.KEP/EC/2021 from the Research Ethics Committee of Padjadjaran University. Written informed consent was obtained from each patient.

Patients who are male, sexually active, aged 17-50 years old, married and have a partner, is an online motorcycle taxi driver who uses a motorcycle as a full-time job; minimum 1 year of working as online motorcycle taxi drivers who are willing to take part in this research; and do not smoke or smoke less than 10 cigarettes per day were included in this study. Patients who are having any history of using drugs to treat ED outside of medical indications; smoke more than 10 cigarettes per day, consume alcohol, had a history of penile and/or testicular surgery or previous urological surgery, history of previous trauma to the penis, pelvis, bones; had a history of systemic disease, and blood vessel disorders such as diabetes mellitus, heart disease, and stroke were excluded from the study.

Data retrieval comes from primary data sources. Respondents who work as online motorcycle taxi drivers and who meet the exclusion and inclusion criteria will be included in this study. The Indonesian version of IIEF-5 was used as a tool to measure ED. The analysis carried out for numerical data to compare the characteristics of this study used Mann–Whitney test and Spearman test.

## Results

This study included 232 respondents. Of the 232 respondents, 149 respondents (64.2%) met the research criteria and 83 respondents (35.8%) did not meet the research criteria. A total of 65 respondents (28%) did not meet the study criteria because of a history of smoking more than 10 cigarettes per day. All research subjects work as online motorcycle taxis as their main job. The mean age in the study was 34.6 ± 7.4 years.

Based on Table 1, the average distance traveled is 84.66 ± 49.401 km/day. The average driving time is 9.6 ± 2.581 hours and the average duration of work is 3.46 ± 1.276 years. In this study, the prevalence of ED was 57.72%. Most of the respondents had mild-moderate ED (IIEF 12-16), which was 37.21% of all respondents who experienced ED.

From Table 2, it is found that the distance traveled (km) has a significant difference with the incidence of ED (*P* = .050). The duration of driving in hours had no significant difference with the incidence of ED(*P* = .888). It was found that there was a significant difference between the duration of work (years) and the incidence of erectile dysfunction (*P* = .045).

Based on the Spearman correlation test (Table 3), it was found that the distance traveled (km) had a negative and significant correlation with the IIEF-5 score (*P* = .024; *r* = −0.098). This shows that the longer distance traveled per day is correlated with the more severe ED as assessed from the IIEF-5 score. Driving time (hours) although had a negative correlation with the IIEF-5 score, it was not statistically significant (*P* = .797; *r* = −0.021). Duration of work (years) had a negative and significant correlation with the IIEF-5 score (*P* = .038; *r* = −0.145). This shows that the longer the subject has worked as an online motorcycle taxi, it is correlated with the more severe the ED as assessed from the IIEF-5 score.

## Discussion

In this study, it was found that the prevalence of ED in online motorcycle taxi drivers who use motorbikes is 57.7%. Another study conducted by Wasike^6^ found that the prevalence of ED among motorcyclists in Kenya was 35.7%. In a study conducted by Naya et al^7^ in Japan, the prevalence of ED in the motorcycle user group was 25%. Another study conducted by Idung et al^8^ showed that the prevalence of ED was 57.5%. This difference may be due to differences in population characteristics in Indonesia, where there are 106 million motorcycle users with most of them using motorcycle as means of daily transportation and also as motorbike taxi drivers. Then, there are differences in the inclusion and exclusion criteria in each study related to the risk factors for ED, the selected sample, and the research method used.

This study found a significant relationship between distance traveled and the incidence and severity of erectile dysfunction (*P* = .050) (Table 2). This is in accordance with a study conducted by Mugalo et al^9^ who examined the relationship between ED in the cycling population and reported that a sample who cycled more than 130 km/week has shown an increased relative risk of ED by 0.9 (0.2-2.4, 95% CI). In another study conducted by Tampubolon et al^10^, a population who cycled more than 400 km/week has shown an increased risk of ED by 0.8 (0.26-2.919, 95% CI). Andersen et al reported a 13% incidence of ED in 160 cyclists who cycled a distance of 540 km. In the study, ED persisted for more than a week and more than a month by 6.9% and 1.9%, respectively. Andersen and Bovin^11^ reported the incidence of impotence and neuropathy (of the genitals and hands) in tour participants who cycled for ± 324 miles. 

This study also has a negative correlation (*P* = .024; *r* = −0.098) (Table 3), meaning that the farther the distance traveled, the lower the IIEF score of −5, causing a higher incidence of ED. Farther distance traveled will be associated with a longer and continuous duration of motorcycle use, causing the duration of compression of the pudendal nerve to occur for a longer period of time.^8^ The effects of compression and vibration can disrupt blood flow in the perineal area and contribute to insufficiency of arterial vascularization to the penis manifesting ED.^12^ Perineal nerve damage may also play a role in the development of ED. Patients with ED have elevated Extracellular Signal Regulated Kinase (ERK) and Endothelin-1 (ET-1) that are associated with the severity of ED (assigned by changes in cavernous blood Growth Hormone (GH), Nitrite oxide (NO), and Cyclic guanosine monophosphate (cGMP) levels).^13^

In this study, it was found that there was no statistically significant difference between the driving duration in hours and the incidence and severity of ED (*P* = .888) (Table 2). This is contrary to the study by Idung^8^ who found that longer motorbike riding was significantly associated with an increase in ED. Based on a study conducted by Wasike et al.^6^ driving duration of 20-39 hours/week increased the incidence of severe ED by 42.9%. In another study conducted by William,^14^ cycling more than 3 hours/week is a risk factor that increases the incidence of ED.^14^ However, in this study, there were no significant findings between the driving duration and the incidence of ED. This can be caused by the online motorcycle taxi work system that is on-demand and can be called at any time so that during this time, online motorcycle taxi drivers do not continue to carry out motorcycle riding activities. One of the hypotheses regarding the relationship between the occurrence of ED with motorbikes is that long-term and continuous use causes nerve damage to the pudendal nerve branches.^15^

In this study, ED also associated with the length of time the subject worked as an online motorcycle taxi driver with significant results (*P *= .045). The results of this study are in accordance to research by Ochiai,^2^ who found that in the population who have ridden a motorcycle for more than 10 years, the incidence of ED increased by 73% compared to those under 10 years by 64%. In another study conducted by Wasike,^6^ driving duration of more than 10 months increased the incidence of ED and the severity of ED related to driving duration. ^8^

In this study, it was found that the length of work (years) had a negative correlation with a score of IIEF-5 (*P* = .038; *r* = −0.145) (Table 3), meaning that the longer the respondent worked as an online motorcycle taxi driver, the lower the IIEF score −5 and the higher risk of ED. This is related to the longer and continuous use of motorbikes, causing the duration of compression of the pudendal nerve to occur for a longer period of time.^8^

The inability to achieve erection during sexual intercourse occurred in 53.78% of respondents, assessed from respondents not getting an erection at all until they only managed to get an erection as much as half of the frequency of sexual intercourse during the last 4 weeks. A total of 52.19% of respondents were unable to perform sexual penetration, obtained from the answers of respondents who could not penetrate at all so that they only managed to penetrate half as much as the frequency of sexual intercourse during the last 4 weeks. Difficulty maintaining an erection occurred in 73.57% of respondents assessed from the answers of respondents who found it difficult, very difficult, to very difficult to maintain an erection in the last 4 weeks.

Motorbike taxi drivers spent many hours a day constantly traveling long distance on a motorbike, which cause repeated compression of the perineum leading to occlusion of the pudendal artery and pudendal nerve. Furthermore, people who have a history of riding a bicycle for more than 3 hours/week can suffer nervous disorders that lead to ED^3,4.^

This study has several limitations. It is a cross-sectional study so no control group was analyzed in this study and the need for research on other work subjects because of the large number of motorcyclists in Indonesia and their diverse uses. It is important to investigate the different types of motorcycles used and its relationship with ED, also, we did not have any data about the subjects’ psychological state regarding ED.

In conclusion, the use of motorbikes can be associated with the risk of developing ED in men. The higher the distance traveled (km), the more severe the DE level found. The longer the duration of work (years), the more severe the level of ED found. There is no significant correlation between the driving duration (hours) and the severity of erectile dysfunction based on the IIEF-5 score for online motorcycle taxi drivers.

## Figures and Tables

**Table 1.** Characteristics of Research Subjects

Characteristics	Total	%
Age (years)		
Mean ± standard deviation	34.6 ± 7.4	
Median (min-max)	34.64 (20-50)	
Education		
Elementary	25	10.77
Junior high	39	16.81
Senior high	147	63.37
University	21	9.05
Distance traveled (km/day)		
Mean ± standard deviation	84.66 ± 49.401	
Median (min-max)	80 (10-250)	
Driving time (hours/day)		
Mean ± standard deviation	9.6 ± 2.581	
Median (min-max)	10 (1-15)	
Duration of work (years)		
Mean ± standard deviation	3.45 ± 1.587	
Median (min-max)	3 (1-6)	
Incidence of ED		
ED	86	57.72
Not ED	63	42.28
ED Severity (IIEF-5)		
Mild (score 17-21)	20	23.26
Mild-moderate (score 12 -16)	32	37.21
Moderate (score 8-11)	30	34.88
Severe (score 5-7)	4	4.65

ED, erectile dysfunction.

**Table 2.** Differences in Distance Traveled, Duration of Driving, and Duration of Work Between Online Motorcycle Taxi Drivers Who Experience ED Compared to Those Who Do Not Experience ED

	ED	Not ED	*P*
Distance traveled (km)			
Mean ± standard deviation	90.79 ± 48.974	76.3 ± 49.15	.050*
Median (min-max)	90 (10-250)	55 (10-200)	
Driving time (hours)			
Mean ± standard deviation	9.63 ± 2.521	9.56 ± 2.681	.888
Median (min-max)	10 (3-15)	10 (1-15)	
Duration of work (years)			
Mean ± standard deviation	3.60 ± 1.295	3.24 ± 1.273	.045*
Median (min-max)	3.5 (1-6)	3 (1-5)	

ED, erectile dysfunction.

*means statistically significant with *P* < = 0.05

**Table 3.** Correlation Between Distance Traveled, Duration of Driving, and Duration of Work with IIEF-5 Scores on Online Motorcycle Taxi Drivers

Variables	Median	IIEF-5	*P*	Spearman
Distance traveled (km)	80 (10-250)	19 (5-25)	.024*	−0.098
Duration of driving (hours/day)	10 (1-15)	19 ( 5-25)	.797	−0.021
Duration of work (years)	3 (1-6)	19 (5-25)	.038*	−0.145

IIEF-5, International Index of Erectile Function–5.

*means statistically significant with *P* < = 0.05

## References

[1] Feldman HA Goldstein I Hatzichristou DG Krane RJ McKinlay JB . Impotence and its medical and psychosocial correlates: results of the Massachusetts Male Aging Study. J Urol. 1994;151(1):54 61. (10.1016/s0022-5347(17)34871-1) 8254833

[2] Ochiai A Naya Y Soh J et al. Do motorcyclists have erectile dysfunction? A preliminary study. Int J Impot Res. 2006;18(4):396 399. (10.1038/sj.ijir.3901445) 16452997

[3] Baran C Mitchell GC Hellstrom WJG . Cycling-related sexual dysfunction in men and women: a review. Sex Med Rev. 2014;2(3-4):93 101. (10.1002/smrj.32) 27784566

[4] Yafi FA Jenkins L Albersen M et al. Erectile dysfunction. Nat Rev Dis Primers. 2016;2:16003. (10.1038/nrdp.2016.3) PMC5027992 27188339

[5] Indonesia BPS. Perkembangan Jumlah Kendaraan Bermotor Menurut Jenis. 1949-2018. Indonesia.

[6] Wasike IW Cheserem E Kagema F . Evaluation of erectile dysfunction among bicycle taxi (Boda Boda) riders in Bungoma Town, Kenya. East Afr Med J. 2017;94(3):193 200.

[7] Schrader SM Breitenstein MJ Lowe BD . Cutting off the nose to save the penis. J Sex Med. 2008;5(8):1932 1940. (10.1111/j.1743-6109.2008.00867.x) 18466268

[8] Idung A Okokon IB Udoh S . Motorcycling as a risk factor for erectile dysfunction: implications for appropriate intervention and prevention strategies. Niger J Fam Pract. 2013;3(2):33 40.

[9] Mugalo EL Ojiambo RM Tam C Erickson B Ayuku D Anjila EL . Occupational cycling is a risk factor for erectile dysfunction in east Africa. East Afr Med J. 2017;94(2):68 71.

[10] Tampubolon DT Wibisono DS Muttaqin Z Julianto HP . Factors influencing the degree of erectile dysfunction in bicycle race athletes. Indones J Urol. 2021;28(1):44 47. (10.32421/juri.v28i1.648)

[11] Andersen KV Bovim G . Impotence and nerve entrapment in long distance amateur cyclists. Acta Neurol Scand. 1997;95(4):233 240. (10.1111/j.1600-0404.1997.tb00104.x) 9150814

[12] Dettori JR Koepsell TD Cummings P Corman JM . Erectile dysfunction after a long-distance cycling event: associations with bicycle characteristics. J Urol. 2004;172(2):637 641. (10.1097/01.ju.0000130749.37731.9f) 15247750

[13] Hamed EA Meki ARMA Gaafar AAA Hamed SA . Role of some vasoactive mediators in patients with erectile dysfunction: their relationship with angiotensin-converting enzyme and growth hormone. Int J Impot Res. 2003;15(6):418 425. (10.1038/sj.ijir.3901059) 14671660

[14] Brant WO Lue TF Smith JF . Does bicycling contribute to erectile dysfunction? Examining the evidence. Phys Sportsmed. 2009;37(1):44 53. (10.3810/psm.2009.04.1682) 20048487

[15] White CR Haidekker MA Stevens HY Frangos JA . Extracellular signal-regulated kinase activation and endothelin-1 production in human endothelial cells exposed to vibration. J Physiol. 2004;555(2):565 572. (10.1113/jphysiol.2003.059899) 14724194 PMC1664844

